# Developing robust quantitative PCR primers for comparative biomass analysis of Tall Fescue (Festuca arundinacea) and its Epichloë endophyte

**DOI:** 10.17912/micropub.biology.001275

**Published:** 2024-12-06

**Authors:** Darrian Talamantes, Caitlin Kirkpatrick, Jason Wallace

**Affiliations:** 1 Institute of Bioinformatics, University of Georgia, Athens, GA, United States; 2 School of Medicine, Emory University, Atlanta, Georgia, United States; 3 Department of Crop and Soil Sciences, University of Georgia, Athens, GA, United States

## Abstract

Tall fescue ( *Festuca arundinacea* ) is a widely adopted forage and turf grass. This is partly due to a fungal endophyte, *Epichloë coenophiala,* which confers both abiotic and biotic stress tolerance. Although PCR primers exist to test for endophyte presence, these were not designed to quantitatively analyze the amount of fungus in the plant. In this study, we test different primer sets for quantitative biomass analysis of tall fescue and *E. coenophiala.* We report standard curves, r-squared, and efficiency values for every primer set and identify those most suited for qPCR in this system.

**Figure 1. Log DNA Concentration vs CT Values of all primers:**
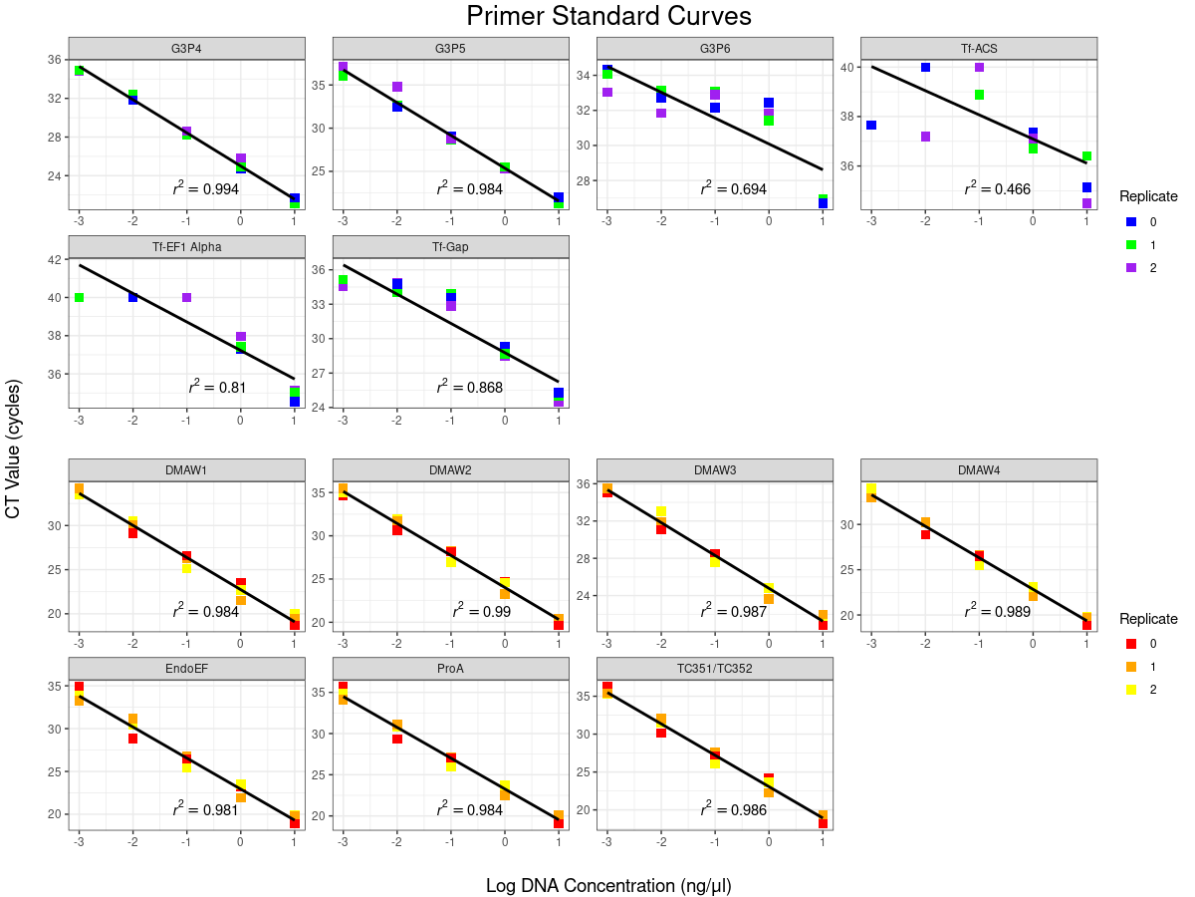
The CT values for each primer set at each DNA concentration are shown, with individual replicates indicated by color. Cool colors (top two rows) are for tall fescue primers, while warm colors (bottom two rows) are for *Epichloë* primers. Only G3P4 and G3P5 have acceptable amplification for tall fescue, while all primer sets for *Epichloë* are acceptable.

## Description

Tall fescue is a cool-season grass that currently covers 14.2 million hectares of land in the Eastern United States (Hmielowski, 2016) **;** it is also used widely in Australia, New Zealand, and Europe (Repussard et al., 2014; Saikkonen et al., 2016) **.** Tall fescue is widely planted as both forage and turf because it is very resilient to a variety of environmental stresses. Many of the advantages of tall fescue are due to a fungal endophyte, *Epichloë coenophiala* . Plants with *Epichloë* endophytes can better resist drought (Nagabhyru et al., 2013) , insects (Clay, 1991) , and nematodes (Elmi et al., 2000) , and also show increased vigor (Clay, 1990) . However, the most common strain of *E. coenophiala* causes “fescue toxicosis” in cattle, with symptoms including shaggy coats, poor weight gain, poor calving and milk production, and gangrene-like symptoms in extremities, all of which result in lost profits for farmers (Kallenbach, 2015) . “Novel” *Epichloë* endophytes that provide stress tolerance without harming livestock have been introduced into elite tall fescue varieties (Realini et al., 2005) , but these are not yet widespread.

The presence or absence of *Epichloë* in tall fescue can be determined either by immunoblot (Hiatt III et al., 1999) or PCR (Young et al., 2015) . While these tests are reliable for determining the presence of *Epichloë* , they are not quantitative. One method for quantitatively determining the amount of *Epichloë* present in a sample is quantitative PCR (qPCR), where the relative amounts of fungal and plant DNA are used as proxies for relative biomass (Tellenbach et al., 2010; Young et al., 2005) . However, when we attempted to use primers from the literature for this purpose (Amombo et al., 2018; Charlton et al., 2012) , we found many of them to be unsuitable for quantitative analysis ( Figure 1 ).

To fill this gap, we designed and tested a series of qPCR primers for both tall fescue and *Epichloë coenophiala* . Primers for tall fescue targeted the glyceraldehyde-3-phosphate dehydrogenase gene (Genbank: GQ480772; primer sets G3P4, G3P5, and G3P6). We compared these with primers from the literature, specifically, Tf-ACS which targets acetyl-CoA synthetase (Amombo et al., 2018) ; Tf-Gap, which targets glyceraldehyde-3-phosphate dehydrogenase (Charlton et al., 2012) ; and Tf-EF1-1α, which targets translation elongation factor 1 alpha (Amombo et al., 2018) . Primers for *Epichloë* targeted the DmaW gene (GeneBank: KX712371.1; primer sets DMA1 through DMA4) (Ekanayake et al., 2017) , which encodes dimethylallyl-tryptophan synthase, the enzyme that catalyzes the first committed step of ergot alkaloid biosynthesis (Ekanayake et al., 2017) . We compared these sets to published primers, namely, ProA.5/ProA.1, which targets the ProA transcriptional regulator sequence (Tanaka et al., 2013) ; TC351/TC352, which targets the DmaW promoter region (Chujo & Scott, 2014) ; and Endo-EF1 which targets *Epichloë* ’s translation elongation factor 1 alpha (Charlton et al., 2012) .

Purified tall fescue and *Epichloë* DNA were diluted to make standard curves for their respective primer sets, which were then tested in triplicate ( Figure 1 ; Table 1). Most tall fescue primers performed poorly. Only G3P4 and G3P5 had r ^2^ values above 0.95 and only G3P5 had an efficiency value above 0.8. In contrast, all *Epichloë* primer sets performed well, with r ^2^ values above 0.95 and all except Endo-EF showing efficiencies above 0.85.

From these results, we conclude that the G3P4 and G3P5 primer sets are the most effective for tall fescue, while any of the tested sets will work for *Epichloë* . The latter is particularly useful since it allows researchers to tailor their amplicon to traits of interest: Endo-EF1 and ProA.5/ProA.1 for general endophyte presence, and the rest to check for ergot alkaloid biosynthesis capability.

## Methods

New primer sets for both tall fescue and *Epichloë coenophiala* were created with Primer3 (Untergasser et al., 2012) . Purified DNA for *Epichloë coenophiala* was isolated by growing hypha in potato dextrose broth with 200 μg/mL of streptomycin, 100 μg/ml of ampicillin and 8 μg/ml of chloramphenicol on an orbital shaker at room temperature (approximately 23° C) for 8 days at 60 rpm. (The concentrations of streptomycin and chloramphenicol were lower than their intended 500 μg/mL and 40 μg/mL due to a miscalculation by the student making the media; since the culture remained clear of bacterial growth it was still used as planned.) Hyphae were strained from the broth with cheesecloth and lyophilized, and DNA was extracted with a Zymo Quick-DNA Fungal/Bacterial miniprep kit (Zymo #D3024) according to the manufacturer’s instructions. Purified DNA was diluted to 10 ng/μl in nuclease-free water, and a set of five 10-fold dilutions were made to be used as qPCR standards. Purified DNA for tall fescue was made from the bottom 2.5 cm of tillers of an endophyte-free plant that was freeze-dried, extracted, and diluted for standards the same way.

All qPCR reactions used a SYBR Green master mix (Roche 04707516001) and were run on a Roche 480 II machine with the following qPCR settings: pre-incubation at 95°C for 5 minutes, followed by 45 cycles of 95°C for 10 seconds, 50°C for 15 seconds, and 72°C for 16 seconds. CT (crossing threshold) values were determined using the instrument software. Primer r ^2^ and efficiency values were calculated in R using the CT values and log-transformed DNA concentration for each sample.

## Reagents

**Table:** 

Primer names	Primer Sequences	r ^2^	Efficiency	Targets	Amplicon Length (bp)	Primer Origin
Tf-ACS	ACCGCGTTCACGTTGTTTTGCCACAACCATGTCGTC	0.466	9.508	Tall Fescue	Unknown ^*^	Amombo et al., 2018
Tf-Gap	CTCAAGGGCATTTTGGGTTATTTCAGAGCAATCCCAGCCTT	0.694	3.776	Tall Fescue	~200 ^†^	Charlton et al., 2012
Tf-EF1-1α	ATGGGTAAGGAAGACAAGACGGAGGTACCAGTGATCATGTT	0.81	3.671	Tall Fescue	~1000 ^†^	Amombo et al., 2018
G3P4	TCGATGAGGACCTTGTTTCCGCTGTATCCCCACTCGTTGT	0.972	0.774	Tall Fescue	134	Novel
G3P5	AGGAGGAGTCTGAGGGTAAGAAGTTGTCGTTCAGAGCAAT	0.984	0.834	Tall Fescue	133	Novel
G3P6	CTTAACGGAAAGTTGACAGGCTTACCCTCAGACTCCTCCT	0.868	1.468	Tall Fescue	138	Novel
DMAW1	GTCCGTCGCAACTGGTAAATTTGTGACTGTCATCCGTGGT	0.984	0.882	Epichloë	153	Novel
DMAW2	GGATTGGAGATGATCCGAGAAAGTTGCTCATTGGGAATGG	0.99	0.863	Epichloë	108	Novel
DMAW3	CATGATTACGAAGCCCTGAATGGCCATTTACCAGTTTCAA	0.987	0.922	Epichloë	111	Novel
DMAW4	CCCGCATCATGATTACGAACTGGCCATTTACCAGTTTCAA	0.989	0.938	Epichloë	119	Novel
ProA.5/ProA.1	GCAGTGGAAACTCGAAATCGGCACTTCTTTCGTCTCAATC	0.984	0.851	Epichloë	186	Tanaka et al., 2013
TC351/TC352	GAGCGCAAAGGTGACTTGTTAAGAAGAGGACGAGCGGAAT	0.985	0.87	Epichloë	90	Chujo & Scott, 2014
Endo-EF1	CGACATTGCCCTCTGGAAGTGGCTTACCAATGACGGTGACA	0.986	0.743	Epichloë	60	Charlton et al., 2012

Notes for table 1: * Tf-ACS did not show a band in gel electrophoresis, and we could not identify targets informatically. † Approximate length based on gel electrophoresis of the PCR product. Primers are named by the gene they target and then a number to distinguish them.
